# Genomic characterization of a dog-mediated rabies outbreak in El Pedregal, Arequipa, Peru

**DOI:** 10.1371/journal.pntd.0012396

**Published:** 2025-03-05

**Authors:** Renzo Salazar, Kirstyn Brunker, Elvis W. Díaz, Edith Zegarra, Ynes Monroy, Gorky N. Baldarrago, Katty Borrini-Mayorí, Micaela De la Puente-León, Natasha Palmalux, Jenna Nichols, Sandeep Kasaragod, Michael Z. Levy, Katie Hampson, Ricardo Castillo-Neyra

**Affiliations:** 1 Zoonotic Disease Research Lab, One Health Unit, School of Public Health and Administration, Universidad Peruana Cayetano Heredia, Lima, Perú; 2 School of Biodiversity, Animal Health and Veterinary Medicine, University of Glasgow, Glasgow, United Kingdom; 3 MRC-University of Glasgow Centre for Virus Research, University of Glasgow, Glasgow, United Kingdom; 4 Laboratorio de Referencia Regional de la Gerencia Regional de Salud de Arequipa, Arequipa, Perú; 5 Red de Salud Arequipa Caylloma, Ministerio de Salud, Arequipa, Perú; 6 Genetic Design and Engineering Center (GDEC), Bioengineering Department, Rice University, Houston, Texas, United States of America; 7 Department of Biostatistics, Epidemiology & Informatics, Perelman School of Medicine, University of Pennsylvania, Philadelphia, Pennsylvania, United States of America; Swiss Tropical and Public Health Institute: Schweizerisches Tropen- und Public Health-Institut, SWITZERLAND

## Abstract

**Background:**

Rabies, a re-emerging zoonosis with the highest known human case fatality rate, has been largely absent from Peru, except for endemic circulation in the Puno region on the Bolivian border and re-emergence in Arequipa City in 2015, where it has persisted. In 2021, an outbreak occurred in the rapidly expanding city of El Pedregal near Arequipa, followed by more cases in 2022 after nearly a year of epidemiological silence. While currently under control, questions persist regarding the origin of the El Pedregal outbreak and implications for maintaining rabies control in Peru.

**Methods:**

We sequenced 25 dog rabies virus (RABV) genomes from the El Pedregal outbreak (n=11) and Arequipa City (n=14) from 2021-2023 using Nanopore sequencing in Peru. Historical genomes from Puno (n=4, 2010-2012) and Arequipa (n=5, 2015-2019), were sequenced using an Illumina approach in the UK. In total, 34 RABV genomes were generated, including archived and newly obtained samples. The genomes were analyzed phylogenetically to understand the outbreak’s context and origins.

**Results:**

Phylogenomic analysis identified two genetic clusters in El Pedregal: 2021 cases stemmed from a single introduction unrelated to Arequipa cases, while the 2022 sequence suggested a new introduction from Arequipa rather than persistence. In relation to canine RABV diversity in Latin America, all new sequences belonged to the new minor clade, Cosmopolitan Am5, sharing relatives from Bolivia, Argentina, and Brazil.

**Conclusion:**

Genomic insights into the El Pedregal outbreak revealed multiple introductions over a 2-year window. Eco-epidemiological conditions, including migratory worker patterns, suggest human-mediated movement drove introductions. Despite outbreak containment, El Pedregal remains at risk of dog-mediated rabies due to ongoing circulation in Arequipa, Puno, and Bolivia. Human-mediated movement of dogs presents a major risk for rabies re-emergence in Peru, jeopardizing regional dog-mediated rabies control. Additional sequence data is needed for comprehensive phylogenetic analyses.

## Author summary

The city of El Pedregal, established in a formerly desert region through an agricultural irrigation project in southern Peru, experienced a dog-mediated rabies outbreak identified in 2021, with cases reported into 2022. Although the outbreak is currently under control, questions about the origin of the El Pedregal outbreak and its implications for maintaining effective dog rabies control in Peru remain unanswered. We examined a re-emerging dog-mediated rabies outbreak in El Pedregal, Peru, using phylogenomic analysis of 34 rabies virus genomes collected from 2010 to 2023. Our results revealed that the 2021 outbreak cases originated from a single introduction unrelated to ongoing rabies in Arequipa, while a 2022 case represented a separate introduction from Arequipa rather than rabies virus persistence within El Pedregal. All new samples fell into a recently identified minor clade (Cosmopolitan Am5), sharing ancestry with rabies virus lineages from Bolivia, Argentina, and Brazil. Our findings suggest that human-mediated dog movement facilitated multiple introductions, and that despite containment efforts, continued circulation in Arequipa, Puno, and Bolivia keeps El Pedregal at risk. Addressing these underlying drivers—particularly the cross-regional movement of dogs—and strengthening genomic surveillance can guide more effective interventions and sustainable rabies control strategies.

## Introduction

Rabies, a globally prevalent zoonotic disease, has one of the highest fatality rates among both humans and animals, with infections primarily resulting from rabid dog bites [1]. The economic burden attributed to rabies surpasses 8 billion USD, with premature death representing a major component [2]. In a concerted effort to eliminate human deaths caused by dog-ediated rabies by 2030, a worldwide strategic plan—‘Zero by 30’—was established in 2015 [3]. To achieve this global goal, it is crucial to have efficient and well-coordinated local surveillance in place [4]. Genomic surveillance, a key tool in molecular epidemiology, provides unique insights into virus dynamics [5,6], spread [7], and control advances [8–10]. In the context of dog-mediated rabies, genomic surveillance can complement epidemiological efforts, offering valuable information to comprehend and redirect control strategies during outbreaks [11].

In Peru, dog-mediated human rabies has been mostly controlled [12,13], but there has been continuous rabies virus (RABV) transmission in the dog population of Arequipa City since its detection in 2015 [14,15]. In 2021, rabid dogs were detected in El Pedregal, a rapidly growing city in the neighboring province of Caylloma, 2 hours travel from Arequipa City by car under typical conditions [16]. El Pedregal was built around an irrigation project in the desert; 40 years ago the area was uninhabited, but it has experienced explosive growth and development [17]. Given its strategic location and economic opportunities, El Pedregal has become a hub for agricultural employment, with considerable in-migration and commuting from neighboring cities and towns [17,18]. The dog rabies outbreak in El Pedregal prompted the local government to implement focused control strategies and strengthen mass dog vaccination campaigns [19]. The outbreak has been controlled, but many epidemiological questions remain unanswered. For instance, the outbreak source and the frequency of introductions are yet unknown, the diversity and distribution of circulating virus lineages have not been characterized, and it is unclear to what extent local transmission may be undetected, all questions that can be answered by genomic surveillance [11,20].

Identifying the source of emerging rabies outbreaks is vital for understanding where dog vaccination needs to be strengthened [21] and understanding the potential for dog-mediated RABV re-emergence. While many countries worldwide contend with endemic dog-mediated rabies virus, the case of El Pedregal presents an instance of re-emergence in a previously rabies-free area (and almost dog-mediated rabies-free continent). This case offers valuable insights into the effectiveness of RABV genomic surveillance and its pivotal role in the latter stages of control efforts. It serves as a warning for potential challenges that may arise in other regions globally, as well as specifically informing the current epidemiological situation in Peru and Latin America, highlighting the evolving epidemiological dynamics of the rabies virus as we strive towards and aim to sustain elimination. Therefore, this study aims to elucidate the origin of the outbreak in El Pedregal using epidemiological and genomic data and to characterize the spread of RABV within this unusual epidemiological context.

## Methods

### Study area

**Fig 1:**
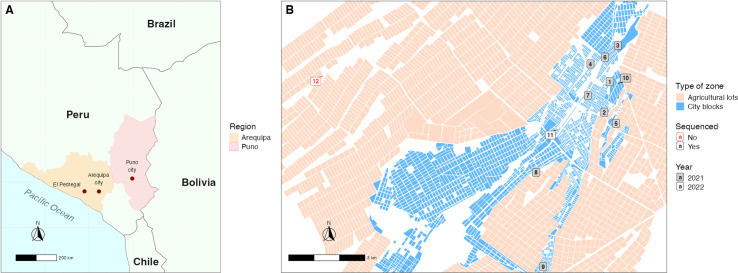
Geographic context of the study area in Peru and zoomed-in map of El Pedregal showing the location of rabies cases during the 2021-2022 outbreak. A) Map of the Southern region of Peru and its borders, with annotations of locations relevant to the outbreak study. B) Detailed map of El Pedregal, showing the locations of rabies cases in rural and urban areas for 2021 and 2022. Cases are numbered according to the epidemiological timeline. The map was drawn in R program. We used the library “ggplot”, and “rnaturalearth” and “rnaturalearthdata”.

This study was conducted in El Pedregal (Fig 1), Majes District, Caylloma Province, Arequipa Department, Peru. Located in the subtropical Coastal desert, approximately 1440 meters above sea level and southwest of Arequipa city [22,23], El Pedregal is characterized by its geographic isolation, surrounded by arid terrain and limited vegetation. Despite being situated in a desert, the area’s unique landscape includes irrigated agricultural zones, which attract human settlement and create conditions conducive to free-roaming dog populations. The proximity to mountainous regions and the presence of established road networks connecting El Pedregal to neighboring areas, including Arequipa City and Puno, facilitate dog translocation and potentially the introduction of RABV.

Human population density in El Pedregal is relatively low compared to urban centers, but because of ongoing in-migration El Pedregal is home to an estimated population of 70,780 inhabitants [24]. The population is mainly composed of permanent migrant populations [17]; up to 90% come from nearby cities and regions such as Arequipa, Puno, and Cusco, as well as neighboring towns [17]. In addition, there is constant flux of seasonal workers from the same regions sometimes with their animals, including dogs. While the diversity of wildlife species in El Pedregal is limited due to its arid desert environment, human-mediated animal inflow—such as the transportation of dogs, livestock, and other domestic animals—plays a significant role in disease dynamics.

### The outbreak

On February 9, 2021, the first case of dog rabies was reported in El Pedregal (Fig 1 and Table 1). The rabid dog was identified when it displayed signs of extreme aggression, entering two homes and biting three people. As a containment measure, the dog was taken to a veterinarian, where rabies was suspected. The dog died on February 10, and the case was laboratory-confirmed on February 11. Following this case, outbreak control activities were conducted by the local health center staff from February 12 to February 15. The surrounding area revealed multiple organic waste dumps and the remains of dead animals, coupled with reports of numerous stray and deceased dogs from neighbors. Fifteen days later, a second case was reported 890 meters away (Fig 1). This second dog bit two random people on the street and the owner reported their dog to the health center. The area where the second case was found was already being monitored and vaccinated due to the activities related to the first positive case. The health inspector observed signs similar to the initial positive case, prompting the owner to choose to euthanize the dog due to suspected rabies, which was confirmed later.

**Table 1:** Epidemiological data collected during dog rabies control activities in El Pedregal.

Case	Month of death	Owned	Owner’s origin	Sex	Age (years)	Accessibility to the street	Last year’s vaccination	Obtention way	Time since acquisition (years)	# of people bitten
1	Feb, 2021	Yes	Puno	F	2.2	Always w/owner	2019	Adopted^*^	NA	2
2	Feb, 2021	Yes	Puno	F	0.6	Half-time	NA	Adopted^*^	0.2	2
3	Mar, 2021	Yes	Huambo, Arequipa	M	1.5	Half-time	2021	Adopted^*^	NA	0
4	Mar, 2021	Yes	NA	M	2.5	Never	2021	Adopted^*^	2.5	0
5	Mar, 2021	Yes	Cuzco	M	2.1	Always	NA	Adopted^*^	0.2	0
6	Mar, 2021	Yes	Pedregal, Arequipa	M	5	Half-time	2021	Adopted^*^	5	0
7	Apr, 2021	Yes	NA	M	5	Always	2021	Bought in market	5	0
8	Jun, 2021	Yes	NA	M	5	Sometimes	NA	Arequipa city	5	0
9	Aug, 2021	No	NA	F	4	Always	NA	Unknown	NA	0
10	Aug, 2021	Yes	NA	M	0.4	Half-time	2021	Bought in countryside	0.4	0
11	Sep, 2022	Yes	NA	M	5	Always w/owner	2019	Adopted^*^	5	1
12^	Oct, 2022	Yes	Pedregal, Arequipa	M	5	Sometimes	2022	Adopted^*^	4.5	0

^Not sequenced;

*People adopt dogs mainly from the streets and from friends/neighbors.

Five days later, during outbreak control activities in the same area, the public health veterinarian from El Pedregal confirmed a third case; seven more cases were detected in the following weeks. Out of these 10 cases, eight were detected within a 1.95 km^2^ area, with the distance between cases ranging from 100 to 1,000 meters. In contrast, cases 8 and 9 were found approximately 3,300 m from the nearest case (Fig 1). Interestingly, six of the ten dog owners with confirmed dog rabies cases sought care in private veterinary clinics, where they received treatment for diseases other than rabies before notifying the public health center. Notably, the area where all the rabies cases were confirmed presented challenges for conducting outbreak control activities during regular working hours (i.e., 8 a.m. to 5 p.m.) due to the absence of owners engaged in agricultural work outside the city, returning home at night. To address this problem, health personnel conducted contact tracing and dog vaccinations during nocturnal hours. Two or three vaccination teams (each team comprising 1 vaccinator and 1 annotator) vaccinated up to 30 dogs daily, with the assistance of public health nurses for contact tracing and an ambulance for mobilization. Some vaccinators were local community members who volunteered and received a 30- to 60-minute training session on rabies transmission and signs, handling dogs, loading syringes, and administering vaccines.

On September 7, 2022, 13 months following the cessation of reported cases, a new rabies incident surfaced in El Pedregal. A five-year-old dog was observed biting a cardboard box and subsequently bit a hen and a 10-year-old girl. Prompt intervention ensued as the family sought the assistance of a trusted veterinarian who helped wash the girl’s wound and evaluated the dog. Following the private veterinarian’s recommendation, the dog was taken to the public health center for further evaluation. Public health centers in Arequipa have veterinarians or sanitary inspectors trained to identify signs of rabies in dogs. They are also equipped to euthanize dogs, collect brain samples, and submit them to the local reference laboratory for analysis. The girl’s bite was classified as mild by an ER physician, and she was started on rabies vaccination and rabies immunoglobulins. Meanwhile, the public health veterinarian at the same center was notified and visited the girl’s house at 9:30 am to evaluate the dog. However, the dog was not present, as it had been sent to a private veterinary clinic for observation, where it died at 11:00 am. A brain sample was extracted and dispatched to the referral laboratory in Arequipa City. The case was not confirmed (by direct Fluorescent Antibody Test (DFAT), see ‘Routine Surveillance in Arequipa’) until September 13, with subsequent control activities prevented by logistical hurdles and owners’ absence during conventional working hours. On September 8, 2022, 33 dogs were vaccinated as part of the presumptive outbreak control measures.

The final instance of rabies in El Pedregal was reported on October 17, 2022, when the owner of the affected dog reported it to the public health center. A private veterinarian, who had been administering the dog treatment for canine distemper since October 15, advised the owner to report the dog after observing neurological signs such as agitation, paralysis, and lethargy. Despite awareness of the mass dog vaccination campaign conducted in July 2022, the owner cited work commitments as hindering the pet’s vaccination, with the dog last receiving rabies vaccination in 2020. Suspecting rabies, the public health center veterinarian euthanized the dog on October 17, took a brain sample, and sent it to the laboratory on the same day, receiving DFAT confirmation of the case on October 18. The distance between these two cases from 2022 was 9,500 m. Subsequent outbreak control measures were implemented on October 19, including vaccination of 24 dogs within the affected vicinity.

Following the detection of the 2021 and 2022 cases, active and passive surveillance activities were conducted in El Pedregal. These included broadcasting public health messages through megaphones mounted on the municipality’s trash trucks and engaging community leaders to raise awareness. Additionally, health personnel conducted active searches for dead dogs, and two public health veterinarians responded to the community reports. In recent years, fieldworkers from the MOH and our research team actively searched for dead dogs and dogs exhibiting neurological signs, collected samples, and submitted them for rabies diagnosis. These searches were carried out in urbanized areas, farming areas, and dumps located outside urban and farming regions. Most of these activities continue to date with varying intensity and no additional cases have been found.

### Rabies viruses

A total of 34 virus samples from DFAT-confirmed rabid dogs in Peru (Arequipa, El Pedregal, Puno) were analyzed in this study. These samples, including 11 outbreak cases from El Pedregal, were obtained from different periods/sources and were sequenced in three distinct subsets. The following describes the samples in the order they were processed:

1. ***Archived RNA from Puno (n=4, 2010-2012):***

Before conducting sequencing in Peru, archived RNA from three dog-associated rabies cases (in livestock) sampled in 2011 and 2012 in Puno, a neighboring rabies-endemic region, were sequenced to obtain whole genome sequences (WGS) with an Illumina metagenomic approach [25] at the Medical Research Council–University of Glasgow Centre for Virus Research (UoG-CVR), Glasgow, UK. Briefly, RNA was Dnase-treated and purified with Zymo quick RNA kit (Zymo Research, R1034) and library preparation was completed using the TruSeq preparation kit (Illumina) following the manufacturer’s instructions, but skipping the Oligo(dT) magnetic bead capture. Samples were fragmented at 94°C for 1 minute (except for one sample subjected to milder fragmentation conditions at 65°C for 5 minutes). Libraries were loaded onto a NextSeq 500 mid output 300 cycle cartridge (2x151), generating approximately 23 million paired end reads in total for the three samples. These samples were collected from cattle with clinical signs of rabies as part of the routine surveillance activities of the National Service for Agrarian Health of Peru (SENASA). RNA extractions were previously partially sequenced as part of a vampire bat rabies surveillance project (GenBank accession nos: KU938752 & KU938829) [26]. The genomes produced here were utilized to design primers for dog variant RABV in Peru (S2 Table) [31] and in later phylogenetic analyses. An additional archived RNA sample from Puno in 2010 obtained from the same source was also sequenced at a later date, as part of batch 2 described below.

1. ***Routine Surveillance in Arequipa (n=5, 2015-2019):***

Five samples collected from routine rabies surveillance in Arequipa between 2015 and 2019 were included in the analysis. For diagnosis, whole brains were extracted and sent to the Regional Reference Laboratory of Arequipa at room temperature in a glycerin-saline solution transport medium. The presence of rabies virus antigen was evaluated using DFAT and confirmed by the mouse inoculation test and RT-PCR at the National Health Institute, following national regulations for rabies diagnosis [27]. These samples were also sequenced with an Illumina metagenomic approach at the UoG-CVR, UK [28]. For these samples, superScript IV Reverse Transcriptase (INVITROGEN 18090010) was used for 1st strand synthesis and NEBNext Ultra II Non-Directional RNA Second Strand Synthesis Module (NEB,E6111) for 2nd strand synthesis. Library preparation from the double-stranded cDNA followed the protocol [28] from step 22, after an Ampure bead clean-up. A concentration of 11pM was loaded onto a MiSeq v2 300 cycle cartridge (2x151 bp), generating 24-30 million paired end reads.

1. ***Outbreak surveillance in El Pedregal (n=11, 2021-2022) and ongoing routine surveillance in Arequipa (n=14, 2021-2023):***

Samples from 11 rabies cases from the El Pedregal outbreak were obtained through local surveillance in this region (between February 2021 – September 2022, Fig 1). These samples underwent the same diagnostic procedures as described above and were sequenced using nanopore sequencing (see below) in the Zoonotic Disease Research Laboratory in Arequipa, Peru. In addition, 14 samples from Arequipa City, collected during the period 2021-2023, were sequenced using the same method.

### Multiplex primer scheme

Whole genomes from the three Puno RABV cases from 2011-12 (sample set 1) were used as references to design a multiplex primer scheme in Primal Scheme [29]. Settings were applied to generate 400 bp products with a 50 bp overlap spanning the entirety of the genome (S2 Table) [31].

### Nanopore sequencing

An established sample-to-sequence protocol was used to extract RNA from brain tissue and generate DNA libraries for nanopore sequencing [30] with the protocol also available from [31]. In brief, RNA was extracted and purified from homogenized brain tissue using a Zymo Quick RNA Miniprep kit with on-column DNase digestion (Zymo Research, USA). A 2-step RT-PCR was performed with RNA reverse transcribed using Lunascript RT Supermix (New England Biolabs, UK) and a multiplex PCR reaction using Q5 High Fidelity Hot-Start DNA Polymerase (New England Biolabs, UK) and the Peru RABV specific multiplex primer set. Library preparation was performed using a Nanopore ligation sequencing kit, SQK-LSK109, with a native barcoding kit, EXP-NBD104/NBD114 (Oxford Nanopore Technologies, UK). Negative controls were included in each run to monitor cross-contamination. The final library was loaded onto an R9.4.1 flow cell and sequenced on a MinION device with live basecalling. Reads were processed using the bioinformatics pipeline described in Bautista, et al. [30] to produce consensus sequences. Any amplicon-specific contamination was masked in the final consensus sequence.

### Whole genome phylogenetic analysis

The 34 WGS generated in this study were aligned with an outgroup sequence, the RABV-GLUE reference sequence for the Cosmopolitan Africa 4 clade (GenBank accession: KF154998), using MAFFT v7.520 [32] with default settings. Phylogenetic tree reconstruction was performed using FastTree v2.1.11 [33] with a gtr+gamma substitution model and local support values obtained using FastTree’s default Shimodaira-Hasegawa test method. The tree was rooted by the outgroup, annotated, and visualized in R [34] v4.3.2 with the ggtree package [35]. Maps were plotted in R using packages rnaturalearth [36] and sf [37,38].

### Contextual phylogenetic analysis

RABV-GLUE [6], a bioinformatics resource for RABV sequence data, was utilized to obtain an alignment and associated metadata of all publicly available canine (excluding bat variant clades) RABV sequences from Latin America (n=1384). Details of these sequences are available in the GitHub repository (https://doi.org/10.5281/zenodo.14282298). The 34 whole genome sequences (WGS) generated in this study were added to this alignment using the ‘add to existing alignment’ function in MAFFT v7.520 [32], ensuring the alignment length was preserved [39]. A phylogenetic tree was produced as described above, using FastTree with annotation/visualization in R, rooted by the same outgroup described above. For comparison, we also undertook the same phylogenetic analysis but using N gene sequences only (whole gene and fragments). For this analysis we discarded sequence data from other parts of the genome, i.e., restricting the newly generated WGSs to their N gene sequences and removing other partial sequences (G=242, L=113, M=75, and P=183).

## Results

We performed a phylogenetic characterization of a dog-mediated rabies outbreak in El Pedregal, analyzing 34 whole genome sequences (WGS) generated in this study, including11 from El Pedregal and 23 from surrounding areas. Additionally, we included a contextual analysis with publicly available dog variant RABV sequences (>200 bp) from the rest of Latin America. This study represents the first whole-genome analysis of dog-mediated RABV in Peru, and indeed in Latin America, with our sequences being the first dog variant rabies virus whole genomes from Peru to be published as available data in the GenBank repository (accession numbers PP965343-PP965374; existing records KU938752 & KU938829 updated to WGS). Comprehensive details, including sequencing platforms, library method, depths of coverage, and epidemiological data such as location, for all new sequences are provided in S1 Table.

### Regional context of study sequences in Latin America

**Fig 2:**
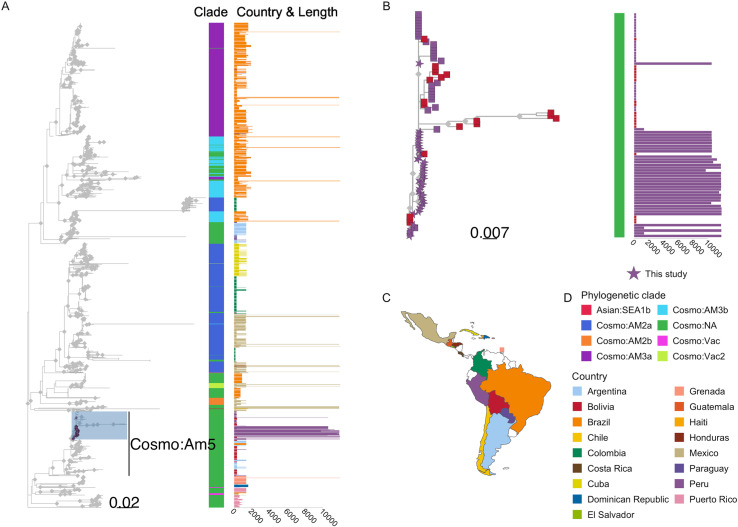
Phylogenetic trees of rabies virus (RABV) sequences from Latin America & the Caribbean (LAC) and newly sequenced genomes from Peru. Scaled in substitutions per site. The map was drawn in R program. We used the library “ggplot”, and “rnaturalearth” and “rnaturalearthdata”. (A) Phylogenetic tree of 1418 RABV sequences from LAC available in NCBI, which include sequences of any length and from any genome region, as well as 34 newly sequenced genomes from El Pedregal, Arequipa, and Puno in Peru. An outgroup (GenBank accession: KF154998) was used to root the tree; the outgroup branch has been excluded for clarity. The sequences from this study are highlighted and new minor clade Cosmo:Am5 is annotated. Color bar I indicates the phylogenetic clade of each sequence and the adjunct bar plot II shows sequence length (base pairs), colored by country of origin; (B) Subtree of the highlighted portion of tree A, showing all descendants of the most recent common ancestor of the Peruvian genomes sequenced in this study and related sequences. Tips are colored according to country of origin, with genomes from this study shown as stars. Color bar I and bar plot II follow the same scheme as in panel A; (C) Map of LAC with the countries of origin for the sequences indicated; (D) Colour schemes and annotation details. Gray diamonds on internal nodes indicate support >80. The scales under each tree indicate the number of substitutions per site.

The current nomenclature for RABV phylogenetic classification is major clade, minor clade, and then lineage, representing increasingly finer genetic resolution (see [6] for detail). On a global scale, Peru RABV sequences, including those from Pedregal and Arequipa city, belong to the Cosmopolitan (Cosmo) major clade. A contextual analysis, including all available RABV sequences (of any length and from any genome region) from Latin America and excluding bat-variant or bat-derived (RAC-SK) sequences, was used to explore the relationship between RABVs from Peru and neighboring countries in detail. Within the Cosmo clade, Peruvian sequences cluster within a large section of the tree that lacks minor clade definition (annotated Cosmo:NA, Fig 2) but clearly stands apart from other known minor clades observed in neighboring countries, delineating at least one new minor clade that we refer here as “Cosmopolitan America 5” (Cosmo:Am5, as previously reported [40] see annotation in Fig 2A). Cosmo:Am5 also includes partial genome sequences (<1354 bp) as previously described [40] from Peru (35 sequences from the period 1985-2012) not from this study, and from Argentina (n=36), Bolivia (n=79) and Brazil (n=5) (Fig 2). The majority (78%) of these partial sequences are very short fragments (~300 bp), with only 26 providing full gene (nucleoprotein) level coverage. Note this excludes a portion of Cosmo:NA sequences from a related part of the tree, below the annotated Cosmo:Am5, which are genetically and geographically distinct (predominantly from island nations).

Within the Cosmo:Am5 group there are further phylogenetic delineations between sequences that indicate the presence of multiple lineages, with possible geographic associations. The subtree of the most recent common ancestor (MRCA) of the Peruvian genomes from this study is expanded in Fig 2B and also contains descendants from Bolivia. For comparison, we provide the phylogenetic tree restricted to N gene sequences as a supplementary figure (S1 Fig). The upper part of the zoomed tree (Fig 2B) contains G gene fragments from Bolivia which are missing from S1 Fig.

### El Pedregal phylogenetic outbreak analysis

**Fig 3:**
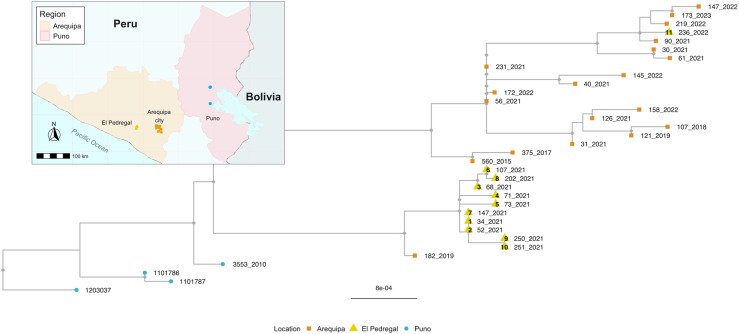
Phylogenetic tree of rabies virus (RABV) whole genome sequences from El Pedregal and surrounding areas. Tips are colored by geographic source, corresponding to the inset map, and labeled with the year of each RABV case. Sequences from El Pedregal (yellow triangles) are labeled with their case identifiers, reflecting the epidemiological timeline of cases and corresponding to Figs 1 and 4 as well as Table 1. An outgroup (GenBank accession: KF154998) was used to root the tree; the outgroup branch has been excluded for clarity, and the scale represents substitutions per site. The map was drawn in R program. We used the library “ggplot”, and “rnaturalearth” and “rnaturalearthdata”.

The Peruvian WGS generated in this study were used to reconstruct a maximum-likelihood phylogeny. There was insufficient temporal signal in the data to enable a molecular clock-based analysis. The samples from Arequipa and Pedregal formed two distinct phylogenetic clusters (Fig 3), each predominantly containing sequences exclusively from their respective area. Ten of the eleven Pedregal sequences collected in 2021 formed one cluster sharing a common ancestor with one Arequipa sequence from 2019, which sits on an orphan branch ancestral to the Pedregal-only cases. The remaining Pedregal sample, representing the 2022 outbreak, clustered amongst all other samples from Arequipa (19 sequences spanning 2018 to 2023).

These results suggest that the El Pedregal 2021 outbreak resulted from a single introduction and brief establishment of local RABV transmission rather than repeated introductions. Given the limited data available, the source of this introduction remains unclear. However, its distinction from Arequipa City cases (except for one isolated case in Arequipa; Fig 3) suggests that it did not come directly from Arequipa but rather that both areas share a common source of introduction, which, considering the ancestral evolutionary history (Fig 3) alongside the epidemiological and demographic context, is likely to be Puno region. In contrast, the El Pedregal 2022 outbreak was caused by a new introduction clearly originating from Arequipa City.

### Epidemiological analysis

**Fig 4:**
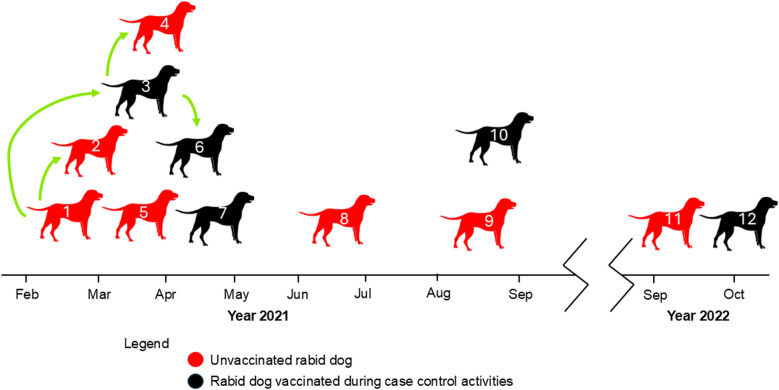
Timeline of dog rabies outbreaks in El Pedregal. The figure highlights the relationship between the cases according to epidemiological data and the reported vaccination status of the rabid dogs. The icons used in this figure were obtained or modified from open-source resources in Openclipart (https://openclipart.org/).

We used the epidemiological data collected during outbreak control activities to reconstruct the timeline of cases in El Pedregal (Fig 4). We inferred a likely transmission chain between case number one with cases two and three (owners were relatives who visited each other with their dogs and lived close to each other); and case number three with cases four and six (owners were acquaintances who visited each other with their dogs). In 2021, seven out of ten cases occurred within the first three months after the index case. It is important to mention that the year before this outbreak the mass vaccination campaign in El Pedregal was canceled due to the pandemic. Thus, all rabid dogs were not vaccinated in the previous year. Interestingly, five of them were vaccinated during the outbreak control activities (Fig 4) but still developed rabies. Among these dogs, the time from vaccination to showing rabies signs was 14, 13, 56, 148, and 1 days. Additionally, the reconstructed timeline shows an epidemiological silence of one year from the last rabid dog reported in 2021 to the next case in 2022 which was the result of a second introduction to El Pedregal based on genetic analysis.

The epidemiological data (Table 1), reveals that most rabid dogs were owned (11/12) and had regular access to the outdoors (at least some hours a week outdoors without restriction) (9/12). Three out of 6 owners were from Puno, and only one was from El Pedregal; we could not obtain this information from the other owners. Dogs’ ages ranged from five months to five years, and three dogs were obtained or adopted from the streets during the outbreak. Additionally, people who were bitten sought treatment for their wounds at the health center between 4 to 7 days after the bite.

## Discussion

In this study, we investigate a dog rabies outbreak in El Pedregal, Arequipa using detailed epidemiological and genomic data.

Our findings reveal that the canine RABV circulating in southern Peru belongs to a novel minor clade within the Cosmopolitan major clade, which we have refer to as “Cosmopolitan Am5,” matching the initial report of an AM5 lineage corresponding to South American AgV1 N gene sequences [40], and in accordance with existing RABV phylogenetic nomenclature [41,42]. This minor clade is distinct from other canine RABV minor clades, including those in neighboring countries. However, our analysis indicates that this clade has been present on the continent since at least 1985, with the first sequence detection in a domestic dog from Lima, Peru (NCBI accession: KF831564).

Notably, most of the sequences from this new minor clade, and LAC sequences in general, are only partial gene or gene level coverage sequences except for the new genomes produced in this study. This limitation makes comparative phylogenetic analyses challenging and restricts the ability to fully utilize genomic data to analyze canine RABV diversity, its circulation, and spread, and to pinpoint external sources of introduction. This lack of sequence data and lack of investment in WGS is likely due to the significant progress in dog rabies elimination in LAC over the last 40 years [43], which coincided with the rise of genome sequencing accessibility and its use as a key surveillance tool [11,44]. Despite these challenges, our analyses demonstrate the value and potential of genomics-informed surveillance to inform dog rabies outbreak response in LAC. Even with a small WGS dataset from our study area, we were able to perform a contextual analysis to identify potential connections with other LAC countries and a fine-scale local analysis that ruled out Arequipa (the most obvious hypothesis) as the source of the initial outbreak in El Pedregal. Taking advantage of newly developed methods [45] to combine the available short sequences in a robust alignment with the WGS from Peru was important for our phylogenetic inference from the contextual dataset. In addition, further research, including collection of data on human-mediated dog movement, as well as genomic surveillance efforts, could inform policies enabling the prevention of future epidemics rather than reactive responses after they occur. Animal movement and the unique geographical and ecological characteristics of El Pedregal collectively underscore the need to incorporate landscape epidemiology into the discussion of rabies control strategies in Arequipa and similar regions.

Our epidemiological data from the 2021 dog RABV outbreak in El Pedregal suggest that two secondary cases can be linked to the index case; one of these secondary cases also produced two new cases of its own. Our WGS phylogenetic analysis supports a local transmission dynamic, showing that all cases sequenced during this period (n=10) clustered together, sharing a common ancestor, and were genetically distinct from the dog RABV circulating in Arequipa City. This supports the hypothesis that a single introduction, followed by local transmission in El Pedregal, was responsible for the 2021 outbreak. The Andean desert around El Pedregal does not harbor any species capable of serving as rabies reservoirs, making human-mediated translocation of infected dogs the most likely mechanism for rabies introductions. This is supported by the high travel intensity between El Pedregal and other endemic regions, such as Puno and Arequipa City.

While our analysis was unable to pinpoint the exact source of introduction for the 2021 outbreak, it was clear from the available data that it did not come directly from Arequipa City, despite a large ongoing dog rabies outbreak in the city for the last eight years and its proximity and continued population interchange with El Pedregal [17,46]. However, a second outbreak in 2022 in El Pedregal did appear to result from an introduction from Arequipa City, rather than the persistence of the first outbreak. Hence, even with limited sequence data El Pedregal demonstrates evidence of at least two introductions within two years, underscoring that introduction and re-emergence is a persistent threat in the region, despite its geographic isolation surrounded by desert. Arequipa City and the Puno region are both potential sources of introduction as neighboring and endemic areas that report active cases of dog rabies [12,47]. Furthermore, the emergence of RABV in Arequipa has previously been linked back to an introduction from Puno [48]. Currently, no measures exist for animal movement control between cities or departments within Peru. Our findings emphasize the need for public awareness campaigns and the implementation of policies to regulate animal movement, which are essential to preventing the introduction and spread of dog rabies in Peru. Moreover, dog vaccination coverage should be maintained in neighboring departments where the risk of introductions remains high.

Our analysis is limited by the small number of rabies cases and sequences available from this outbreak. There were also relatively few historical RABV sequences from Latin America [11], and they were mainly restricted to very short sequences (e.g., partial gene ~200-300 bp), limiting the degree to which we could infer outbreak origins. For future studies, we suggest obtaining genomes from a larger number of samples from Arequipa City and the Puno region over the same period to provide more conclusive evidence of the source of RABV emergence in El Pedregal and facilitate more comprehensive phylodynamic analyses that could elucidate transmission drivers and diffusion pathways.

Across diverse global regions where dog RABV has been studied, incursions of dog RABV appear to be common, with genomic surveillance revealing higher rates than expected [9,21]. Human-mediated movement of dogs has emerged as a significant driver [25,49] of these occurrences. While many introductions fail to establish due to stochastic factors in rabies transmission [50], areas with increased human movement are likely to face heightened risks. This is evident in El Pedregal, which has a history of migratory settlement and where a portion of the population commutes daily, weekly, or seasonally from nearby cities to Pedregal for work [17]. Moreover, poor housing conditions in the peri-urban areas of El Pedregal prevent dog owners from keeping dogs inside homes, making these dogs less accessible and harder to vaccinate, and increasing the vulnerability of El Pedregal to new introductions, similar to the ongoing rabies outbreak in Arequipa City [15,17]. In Arequipa City, such conditions were identified as the main factors that allowed the introduction and persistence of the RABV, together with landscape features, such as dry water channels that allowed unrestricted and fast movement of dogs across large parts of the city, facilitating the rapid spread of the RABV [15,51].

Mass dog vaccination will be crucial to preventing introductions and their onward spread. Before the COVID-19 pandemic, mass dog vaccination coverage in El Pedregal was initially reported as 82-100%, likely due to an underestimation of the dog population. After the 2021 cases and a re-estimation of the dog population, annual mass dog vaccination coverage has been revised and is estimated to be between 47% and 63%. The year before the 2021 outbreak in El Pedregal, mass dog vaccinations were canceled due to the COVID-19 pandemic, providing a window of opportunity for the virus to take hold. Although the outbreak prompted a rapid local response, including dog vaccinations, five of the vaccinated dogs still contracted and died from rabies, even after months of being vaccinated. For most cases, it remains unclear whether the vaccine was administered too late or if it was ineffective. Assessing the efficacy of these ring vaccination activities is crucial. Currently, in Arequipa, when owners of exposed dogs do not permit euthanasia, these dogs are vaccinated in an attempt to prevent new cases. Furthermore, the last reported rabies case in El Pedregal (October 17, 2022) did not receive vaccination during the 2022 outbreak response but had been vaccinated in 2020, according to its owner. While annual boosters are recommended, these observations raise questions about the vaccine efficacy or the administration process (e.g., poorly trained vaccinators might inoculate in the wrong area/tissue). Despite these challenges, it is important to note that in Peru, as in much of the Americas, rabies surveillance, outbreak response, and dog bite case management are integrated within the Ministry of Health’s health system. In regions where health systems and rabies programs differ, outbreak response and surveillance should be adapted to align with local norms and contexts.

Genomic information has the potential to shed light on complex epidemiological scenarios. This study provides a snapshot of RABV introduction in a rapidly urbanizing rural area, a common scenario in Latin American cities. Our results indicate multiple introductions into El Pedregal in 2021 and 2022, most likely mediated by human translocation of dogs. This study highlights the potential of genomics analysis to understand rabies outbreaks, enhance surveillance systems, and inform rabies control efforts.

## Supporting information

S1 Table Sequencing and epidemiology details of newly sequenced rabies virus sequences used in this study. *Genbank records updated. Coding region coverage is indicated according to each gene (N, P, M, G, L).
(XLSX)

S2 Table Details of primers used for amplicon-based sequencing of rabies viruses in Peru. (DOCX)

S1 Fig Phylogenetic trees of rabies virus (RABV) N gene sequences from Latin America & the Caribbean (LAC) including the new sequences from Peru. (A) Phylogenetic tree of 1050 N gene sequences from LAC available in NCBI, which include short N gene fragments, and the N gene sequences from the 34 newly sequenced genomes from El Pedregal, Arequipa, and Puno in Peru. An outgroup (GenBank accession: KF154998) was used to root the tree; the outgroup branch has been excluded for clarity. The sequences from this study are highlighted and new minor clade Cosmo:Am5 is annotated. Color bar I indicates the phylogenetic clade of each sequence and the adjacent bar plot II shows sequence length (base pairs), colored by country of origin; (B) Subtree of the highlighted portion of tree A, showing all descendants of the most recent common ancestor of the Peruvian viruses sequenced in this study and related sequences. Tips are colored according to country of origin, with genomes from this study shown as stars. Color bar I and bar plot II follow the same colour scheme as in panel A. Colour schemes and annotation details are the same as in the main Fig 1. Gray diamonds on internal nodes indicate support >80. Scales under each tree indicate the number of substitutions per site.
(TIF)
