# Supplementary material for: Genomic characterization of a dog-mediated rabies outbreak in El Pedregal, Arequipa, Peru
Source: PLoS Negl Trop Dis. 2025 Mar 5;19(3):e0012396. doi: 10.1371/journal.pntd.0012396 (PMC12043231; doi:10.1371/journal.pntd.0012396)
Supplement: S2 Table — (DOCX) [file pntd.0012396.s002.docx]

**S2 Table.** Details of primers used for amplicon-based sequencing of rabies viruses in Peru.

| Primer Name | Pool | Sequence | Length | Tm | GC% | Start | End |
| --- | --- | --- | --- | --- | --- | --- | --- |
| rabvPeru2_1_LEFT | 1 | CAAAATCAGAGAAGAAGCAGACAGC | 25 | 60.62 | 44 | 11 | 36 |
| rabvPeru2_1_RIGHT | 1 | GCCCAATTCCCTTCCACATCAG | 22 | 61.53 | 54.55 | 432 | 410 |
| rabvPeru2_2_LEFT | 2 | CAGAAGACTGGACCAGCTATGGA | 23 | 61.45 | 52.17 | 326 | 349 |
| rabvPeru2_2_RIGHT | 2 | GTGCCCACTCTGATTGCTGAAT | 22 | 61.2 | 50 | 753 | 731 |
| rabvPeru2_3_LEFT | 1 | TGTGCTAATTGGAGTACTATCCCGA | 25 | 61.16 | 44 | 649 | 674 |
| rabvPeru2_3_RIGHT | 1 | AGGAGCACATGCAGCAATAACC | 22 | 61.53 | 50 | 1075 | 1053 |
| rabvPeru2_4_LEFT | 2 | TGCCGTTGGTCATGTGTTCAAT | 22 | 61.33 | 45.45 | 978 | 1000 |
| rabvPeru2_4_RIGHT | 2 | TTTGGACGGGCTTGATGATTGG | 22 | 61.47 | 50 | 1377 | 1355 |
| rabvPeru2_5_LEFT | 1 | TCCTGAGGCTGTTTATGCTCGA | 22 | 61.47 | 50 | 1272 | 1294 |
| rabvPeru2_5_RIGHT | 1 | AAGTCGTCTCATGTCCTCAGGG | 22 | 61.47 | 54.55 | 1681 | 1659 |
| rabvPeru2_6_LEFT | 2 | AGATGGCCGAAGAGACTGTTGA | 22 | 61.41 | 50 | 1568 | 1590 |
| rabvPeru2_6_RIGHT | 2 | TTCCTGGAGGGTTGGGAAAGTT | 22 | 61.51 | 50 | 1928 | 1906 |
| rabvPeru2_7_LEFT | 1 | TAGTCCGGCAAATGAGGTCAGG | 22 | 62.06 | 54.55 | 1823 | 1845 |
| rabvPeru2_7_RIGHT | 1 | CTGGCACATTTTTCGCCTCCTT | 22 | 61.98 | 50 | 2249 | 2227 |
| rabvPeru2_8_LEFT | 2 | AGTTTCCCTCTCGATCTTCAGGG | 23 | 61.45 | 52.17 | 2153 | 2176 |
| rabvPeru2_8_RIGHT | 2 | CTGACACGGGAGAGGGTTTTTG | 22 | 61.39 | 54.55 | 2565 | 2543 |
| rabvPeru2_9_LEFT | 1 | TCTGAGATGTCCCAGAGTCAACA | 23 | 60.76 | 47.83 | 2443 | 2466 |
| rabvPeru2_9_RIGHT | 1 | ATACCCAGTTCATGCCCTCAGG | 22 | 61.82 | 54.55 | 2835 | 2813 |
| rabvPeru2_10_LEFT | 2 | TGAGATATACTCTGGGAATCATCGGA | 26 | 60.52 | 42.31 | 2734 | 2760 |
| rabvPeru2_10_RIGHT | 2 | GAAGCAGAGAGGAGTCTTTGTCC | 23 | 60.62 | 52.17 | 3096 | 3073 |
| rabvPeru2_11_LEFT | 1 | CCAGGGCAGAATTTGGTGTATCA | 23 | 60.88 | 47.83 | 2986 | 3009 |
| rabvPeru2_11_RIGHT | 1 | AGGGACCAAGTTTGTCCGGTAT | 22 | 61.36 | 50 | 3412 | 3390 |
| rabvPeru2_12_LEFT | 2 | GGAAAGATGGTTCCTCAGGTCC | 22 | 60.36 | 54.55 | 3309 | 3331 |
| rabvPeru2_12_RIGHT | 2 | CCAATGGTAGTCAGGGTACGGA | 22 | 61.22 | 54.55 | 3735 | 3713 |
| rabvPeru2_13_LEFT | 1 | CACAACCACGTTCAAAAGAAAGCA | 24 | 61.14 | 41.67 | 3605 | 3629 |
| rabvPeru2_13_RIGHT | 1 | TCCCTTTGGATGCTCTCTTCCC | 22 | 61.76 | 54.55 | 3982 | 3960 |
| rabvPeru2_14_LEFT | 2 | ACTTACTGCTCAACCAACCACG | 22 | 61.06 | 50 | 3870 | 3892 |
| rabvPeru2_14_RIGHT | 2 | GTGCATCCAGACACTCCTCTCT | 22 | 61.47 | 54.55 | 4231 | 4209 |
| rabvPeru2_15_LEFT | 1 | CCTGATCAGTTGGTGAACCTGC | 22 | 61.45 | 54.55 | 4131 | 4153 |
| rabvPeru2_15_RIGHT | 1 | TGCTGGAGTAGGGATGATTGCA | 22 | 61.49 | 50 | 4520 | 4498 |
| rabvPeru2_16_LEFT | 2 | GGTGTCATCCTCATGTGAACGG | 22 | 61.25 | 54.55 | 4420 | 4442 |
| rabvPeru2_16_RIGHT | 2 | TAGATTCTGGCCGACTGACTCC | 22 | 61.27 | 54.55 | 4780 | 4758 |
| rabvPeru2_17_LEFT | 1 | TGGGGGAAGTATGTATTAATAAGTGCA | 27 | 60.15 | 37.04 | 4680 | 4707 |
| rabvPeru2_17_RIGHT | 1 | GTTGGTCACTGAAACTGCCAGA | 22 | 61 | 50 | 5100 | 5078 |
| rabvPeru2_18_LEFT | 2 | ACAGGGTAGATTTAAGAGTCAAGAGAC | 27 | 60.09 | 40.74 | 4989 | 5016 |
| rabvPeru2_18_RIGHT | 2 | AGATGAGAAGTGTTGCCGGTTG | 22 | 61.13 | 50 | 5392 | 5370 |
| rabvPeru2_19_LEFT | 1 | GCATCTCAGCAAAGTGTACATAACTCT | 27 | 61.53 | 40.74 | 5287 | 5314 |
| rabvPeru2_19_RIGHT | 1 | GCAGTTCCACCCACTTTGAGAG | 22 | 61.39 | 54.55 | 5689 | 5667 |
| rabvPeru2_20_LEFT | 2 | GGATGACTCTGACAGACAACTGC | 23 | 61.17 | 52.17 | 5592 | 5615 |
| rabvPeru2_20_RIGHT | 2 | CTCTTCGTCCCAGTCTAAGGCA | 22 | 61.47 | 54.55 | 5984 | 5962 |
| rabvPeru2_21_LEFT | 1 | AGGCATTTGGAAGGTATCTGGC | 22 | 60.69 | 50 | 5889 | 5911 |
| rabvPeru2_21_RIGHT | 1 | AGCCGGAGTTTCCACACATAGA | 22 | 61.41 | 50 | 6291 | 6269 |
| rabvPeru2_22_LEFT | 2 | TGTCTCGCTTCAACTCCTTAATGA | 24 | 60.02 | 41.67 | 6159 | 6183 |
| rabvPeru2_22_RIGHT | 2 | GTGCCCCCAATGTCTGTAACAG | 22 | 61.46 | 54.55 | 6527 | 6505 |
| rabvPeru2_23_LEFT | 1 | GACTTTCGGTCCCAGTGCAAAA | 22 | 61.58 | 50 | 6424 | 6446 |
| rabvPeru2_23_RIGHT | 1 | GTCCATCGACTCGGGAATCTCA | 22 | 61.59 | 54.55 | 6827 | 6805 |
| rabvPeru2_24_LEFT | 2 | ATCAAAACCCAGACATGGCCAC | 22 | 61.41 | 50 | 6722 | 6744 |
| rabvPeru2_24_RIGHT | 2 | CGTCGAAGAGTGGCAGGATGTA | 22 | 62.09 | 54.55 | 7152 | 7130 |
| rabvPeru2_25_LEFT | 1 | ACGAGAATTGAAGATTGAGGGTCG | 24 | 60.92 | 45.83 | 7039 | 7063 |
| rabvPeru2_25_RIGHT | 1 | CCCATAACCCGATGAGGTCTGA | 22 | 61.28 | 54.55 | 7425 | 7403 |
| rabvPeru2_26_LEFT | 2 | TCATGTGTTTGGATTGAAGAGAGTGT | 26 | 60.91 | 38.46 | 7324 | 7350 |
| rabvPeru2_26_RIGHT | 2 | TCAGCCCTAGTTTAGATGCCCC | 22 | 61.56 | 54.55 | 7734 | 7712 |
| rabvPeru2_27_LEFT | 1 | GTGCTATGTCCGACGTATATGTTGT | 25 | 61.02 | 44 | 7601 | 7626 |
| rabvPeru2_27_RIGHT | 1 | ACTGCCTGTACTGACATGAGCA | 22 | 61.67 | 50 | 7984 | 7962 |
| rabvPeru2_28_LEFT | 2 | GTCGACTGTATCCACCAATGCG | 22 | 61.62 | 54.55 | 7888 | 7910 |
| rabvPeru2_28_RIGHT | 2 | TTTCTCCAAGATCCGGGTTCCC | 22 | 62.02 | 54.55 | 8262 | 8240 |
| rabvPeru2_29_LEFT | 1 | CGTCAGTTCTCAGACCCTGTCT | 22 | 61.4 | 54.55 | 8141 | 8163 |
| rabvPeru2_29_RIGHT | 1 | TGCCTTCTTATTGTCCGGGAGT | 22 | 61.42 | 50 | 8575 | 8553 |
| rabvPeru2_30_LEFT | 2 | TCCCTCGATTTCTCAGTGAGCT | 22 | 60.89 | 50 | 8478 | 8500 |
| rabvPeru2_30_RIGHT | 2 | TTAGGGGGCCTCGTGAGAAAAA | 22 | 61.63 | 50 | 8895 | 8873 |
| rabvPeru2_31_LEFT | 1 | TCCATTTCTTGCGCTTGTGGAG | 22 | 61.71 | 50 | 8792 | 8814 |
| rabvPeru2_31_RIGHT | 1 | ACCCTCCTTCACTGTATCTGGC | 22 | 61.48 | 54.55 | 9165 | 9143 |
| rabvPeru2_32_LEFT | 2 | TGTCTCTGACAGGCCCAGATTT | 22 | 61.36 | 50 | 9060 | 9082 |
| rabvPeru2_32_RIGHT | 2 | GAAGCTTCTGGAAGTGAGGCAC | 22 | 61.45 | 54.55 | 9483 | 9461 |
| rabvPeru2_33_LEFT | 1 | AGGCCCATTGATGATATAACATTGGA | 26 | 60.47 | 38.46 | 9371 | 9397 |
| rabvPeru2_33_RIGHT | 1 | ACCCCTGAGATCAATTCAAGAGGT | 24 | 61.32 | 45.83 | 9766 | 9742 |
| rabvPeru2_34_LEFT | 2 | AGAGACTATTTGAGGGGGCTCG | 22 | 61.28 | 54.55 | 9653 | 9675 |
| rabvPeru2_34_RIGHT | 2 | ACAGGTTTTTCTCAACCCTCTGG | 23 | 60.69 | 47.83 | 10083 | 10060 |
| rabvPeru2_35_LEFT | 1 | ACTGGCTATGGATCTTCTCAGACT | 24 | 60.63 | 45.83 | 9978 | 10002 |
| rabvPeru2_35_RIGHT | 1 | TTCGCTGAGGTAGAGACTGCAA | 22 | 61.4 | 50 | 10354 | 10332 |
| rabvPeru2_36_LEFT | 2 | CAGCTAGAACCATGACCGGAGA | 22 | 61.53 | 54.55 | 10242 | 10264 |
| rabvPeru2_36_RIGHT | 2 | GATGTGTTCCGGAAGCCATCAG | 22 | 61.57 | 54.55 | 10629 | 10607 |
| rabvPeru2_37_LEFT | 1 | GGGGGATATCTAGGGCAGTTCT | 22 | 60.5 | 54.55 | 10530 | 10552 |
| rabvPeru2_37_RIGHT | 1 | TCACGAGCATAGTCCCGTATGT | 22 | 61.01 | 50 | 10917 | 10895 |
| rabvPeru2_38_LEFT | 2 | TGACATTGCATCGATCAACCGG | 22 | 61.57 | 50 | 10810 | 10832 |
| rabvPeru2_38_RIGHT | 2 | AGACTCTACATCACTGTCAATCAGAGT | 27 | 61.23 | 40.74 | 11243 | 11216 |
| rabvPeru2_39_LEFT | 1 | GCAGAGAGCTCGTTCCTTGAAC | 22 | 61.49 | 54.55 | 11128 | 11150 |
| rabvPeru2_39_RIGHT | 1 | TATACAGGTCGTGAAGCCTTGC | 22 | 60.42 | 50 | 11499 | 11477 |
| rabvPeru2_40_LEFT | 2 | TGACCCCAAAATCCTGAGACACT | 23 | 61.53 | 47.83 | 11392 | 11415 |
| rabvPeru2_40_RIGHT | 2 | GGATGTACAGGCTTCTGGGGAT | 22 | 61.56 | 54.55 | 11821 | 11799 |
| rabvPeru2_41_LEFT | 1 | GCAAGTCATTCGAGGGAATATTTACCT | 27 | 61.38 | 40.74 | 11527 | 11554 |
| rabvPeru2_41_RIGHT | 1 | AATCAAACAACCAGAGGCTCGG | 22 | 61.14 | 50 | 11888 | 11866 |
