# Supplementary figures and images for: Genomic characterization of a dog-mediated rabies outbreak in El Pedregal, Arequipa, Peru
Source: PLoS Negl Trop Dis. 2025 Mar 5;19(3):e0012396. doi: 10.1371/journal.pntd.0012396 (PMC12043231; doi:10.1371/journal.pntd.0012396)

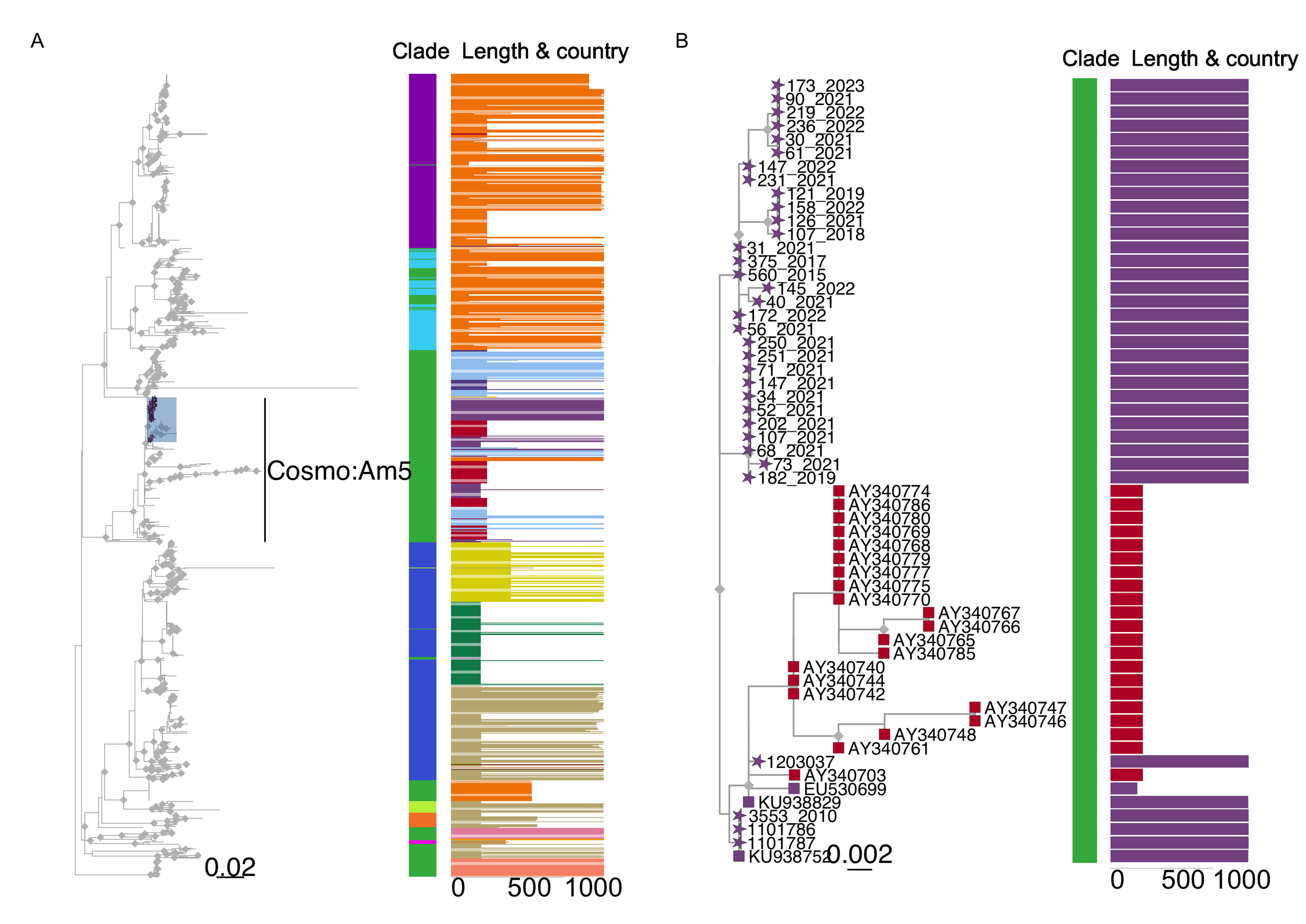

Supplement: S1 Fig — (A) Phylogenetic tree of 1050 N gene sequences from LAC available in NCBI, which include short N gene fragments, and the N gene sequences from the 34 newly sequenced genomes from El Pedregal, Arequipa, and Puno in Peru. An outgroup (GenBank accession: KF154998) was used to root the tree; the outgroup branch has been excluded for clarity. The sequences from this study are highlighted and new minor clade Cosmo:Am5 is annotated. Color bar I indicates the phylogenetic clade of each sequence and the adjacent bar plot II shows sequence length (base pairs), colored by country of origin; (B) Subtree of the highlighted portion of tree A, showing all descendants of the most recent common ancestor of the Peruvian viruses sequenced in this study and related sequences. Tips are colored according to country of origin, with genomes from this study shown as stars. Color bar I and bar plot II follow the same colour scheme as in panel A. Colour schemes and annotation details are the same as in the main Fig 1. Gray diamonds on internal nodes indicate support >80. Scales under each tree indicate the number of substitutions per site. (TIF) [file pntd.0012396.s003.tif]
